# NOD-Like Receptor Protein 3 Inflammasome-Dependent IL-1β Accelerated ConA-Induced Hepatitis

**DOI:** 10.3389/fimmu.2018.00758

**Published:** 2018-04-10

**Authors:** Jingyun Luan, Xuyao Zhang, Shaofei Wang, Yubin Li, Jiajun Fan, Wei Chen, Wenjing Zai, Sijia Wang, Yichen Wang, Mingkuan Chen, Guangxun Meng, Dianwen Ju

**Affiliations:** ^1^Department of Microbiological and Biochemical Pharmacy, The Key Laboratory of Smart Drug Delivery, Ministry of Education, School of Pharmacy, Fudan University, Shanghai, China; ^2^Unit of Innate Immunity, Key Laboratory of Molecular Virology and Immunology, Institute Pasteur of Shanghai, Shanghai Institutes for Biological Sciences, Chinese Academy of Sciences, Shanghai, China

**Keywords:** NOD-like receptor protein 3 inflammasome, IL-1β, caspase-1, pyroptosis, autoimmune hepatitis

## Abstract

Autoimmune hepatitis (AIH) is a progressive inflammatory disorders of unknown etiology, characterized by immune-mediated destruction of hepatocytes and massive production of cytokines. Interleukin-1β is a pleiotropic proinflammatory cytokine and well known to be critical in a variety of autoimmune diseases. However, the role of interleukin-1β (IL-1β) in AIH is still indistinct. Here, we first investigated the significance of NOD-like receptor protein 3 (NLRP3) inflammasome-dependent IL-1β in the pathogenesis of AIH with a murine model of immune-mediated hepatitis induced by Concanavalin A (ConA). In ConA-treated mice, pathogenic elevated NLRP3, Cleaved caspase-1 and IL-1β levels, as well as an inflammatory cell death known as pyroptosis predominantly occurred in the livers. Strikingly, *NLRP3^−/−^* and *caspase-1^−/−^* mice were broadly protected from hepatitis as determined by decreased histological liver injury, serum aminotransferase (ALT)/aspartate transaminase levels, and pyroptosis. *In vivo* intervention with recombinant human interleukin-1 receptor antagonist (rhIL-1Ra) strongly suppressed ConA-induced hepatitis by decreasing tumor necrosis factor-alpha (TNF-α) and interleukin-17 (IL-17) secretion, and inflammatory cell infiltration into livers. Additionally, rhIL-1Ra-pretreated mice developed significantly reduced NLRP3 inflammasome activation and reactive oxygen species (ROS) generation. Scavenging of ROS by *N*-acetyl-cysteine also attenuated NLRP3 inflammasome activation and liver inflammation, indicating that the essential role of ROS in mediating NLRP3 inflammasome activation in ConA-induced hepatitis. In conclusion, our results demonstrated that NLRP3 inflammasome-dependent IL-1β production was crucial in the pathogenesis of ConA-induced hepatitis, which shed light on the development of promising therapeutic strategies for AIH by blocking NLRP3 inflammasome and IL-1β.

## Introduction

Autoimmune hepatitis (AIH) is a progressive inflammatory disorder that may start with an acute hepatitis and result in liver cirrhosis, fulminant hepatic failure, or liver cancer (1–4). Although the etiology of AIH is not fully understood, emerging evidence indicates that proinflammatory cytokines, such as tumor necrosis factor-alpha (TNF-α), interferon-γ (IFN-γ), and interleukin-17 (IL-17), are known mediators regulating inflammatory dysregulation in AIH (5–9). Data from clinical studies have drawn attention that anti-TNF-α (infliximab) antibody shows efficacies as a rescue therapy in some patients with AIH, but displays high risks of severe adverse events, especially infections (10, 11). It thus seems that further research is needed to identify some other potential proinflammatory cytokines in AIH.

The pleiotropic proinflammatory cytokine, interleukin-1β (IL-1β), costimulates T lymphocytes, induces Th17 differentiation, and recruits inflammatory cell, thereby promoting numbers of autoimmune diseases (12). Recent clinical studies demonstrated that the expression of IL-1β in patients with AIH was significantly increased and correlated with aggravation of hepatitis (7). IL-1β, together with IFN-γ and TNF-α, enhances intercellular cell adhesion molecule-1 expression in hepatocytes, thus contributing to development of AIH (13). Additionally, IL-1β has been demonstrated essential for the differentiation of Th0 lymphocytes into Th17 cells, which has been considered as an important target for AIH by secreting IL-17 and suppressing regulatory T cells (Tregs) (7, 14). These findings suggest a potential important role of IL-1β in the development of AIH.

NOD-like receptor protein 3 (NLRP3) inflammasome, a multiprotein complex, contributes to the development of liver disorders, such as alcoholic steatohepatitis, viral fulminant hepatitis, and non-alcoholic fatty liver disease, through processing caspase-1 cleavage and IL-1β secretion (15–18). Upon sensing noxious signals, NLRP3 recruits pro-caspase-1 and the adaptor molecule ASC (apoptotic specklike protein-containing CARD), resulting in activation of caspase-1 as well as secretion of IL-1β and interleukin-18 (IL-18), which play a critical role in promoting liver inflammation (19). NLRP3 inflammasome can be activated by a variety of danger sensors, such as excess ATP, uric acid, and mitochondrion DNA, while mitochondria-derived reactive oxygen species (mtROS) was considered as a major activator (20–23). Interestingly, a study recently illustrated that Concanavalin A (ConA)-induced hepatitis was accompanied with reactive oxygen species (ROS) production (24). However, it remains unknown whether ROS generation could activate NLRP3 inflammasome and lead to IL-1β production in AIH.

ConA-induced hepatitis is characterized by T lymphocytes activation and massive pro-inflammatory cytokines production as well as hepatocyte death in the livers, thereby mimicking patterns of AIH (25–27). In the present study, we explored the effect of IL-1β in ConA-induced hepatitis, particularly the mechanism of its activation, which was dependent on NLRP3 inflammasome. In addition, the potential therapeutic efficacy of recombinant human IL-1Ra (rhIL-1Ra) and its regulation mechanism were also evaluated. Results from this study shed light on the development of novel promising therapeutic strategies for AIH.

## Materials and Methods

### Materials

Recombinant human interleukin-1 receptor antagonist (WYP1001) was provided by General Regeneratives Limited (Shanghai, China). ConA (C2010) was purchased from Sigma-Aldrich (St. Louis, MO, USA). ROS scavenger *N*-acetyl-cysteine (NAC) (S0077) was obtained from Beyotime (Haimen, China). The primary antibodies used for western blot, including antibodies to NLRP3 (15101), Cleaved caspase-1 (67314), IL-1β (12242), GAPDH (51332), JAK2 (3230), p-JAK2 (Tyr1007/1008) (3776), STAT3 (4904), and p-STAT3 (Tyr705) (91315), were purchased from Cell Signaling Technology (Danvers, MA, USA). The secondary antibodies (MR-R100 and MR-M100) used for western blot were obtained from MR Biotech (Shanghai, China). The primary antibody of NLRP3 (ab4207) and donkey anti-goat IgG (ab150131) used for immunofluorescence staining were purchased from Abcam (Cambridge, UK). The antibodies against CD11b (ab133357) and CD68 (ab955) were obtained from Abcam (Cambridge, UK).

### Animal Models

Six to eight weeks old BALB/c mice were purchased from Shanghai SLAC laboratory animal Co. Ltd. (Shanghai, China). *NLRP3^−/−^* mice had been described before (28, 29). *Caspase-1^−/−^* mice were obtained from Jackson Laboratory. Both *NLRP3^−/−^* and *caspase-1^−/−^* mice had been crossed with BALB/c mice for 10 generations for the current project. To establish the murine model of AIH, ConA was injected by tail vein at a dose of 20 mg/kg body weight. RhIL-1Ra was given intraperitoneally at 20 mg/kg every 6 for 24 h before ConA injection, and the treatment with rhIL-1Ra was ongoing until sacrifice. NAC was intraperitoneally administrated 2 h before ConA injection at a dosage of 0.6 mg/kg. Mice were sacrificed at indicated time points for sampling of serum and liver. Blood obtained from orbital sinus was collected and stand for 1 h. The serum was separated by centrifuging at 3,000 rpm for 10 min at 4°C and used for the detection of liver function and cytokine levels.

### Liver Histology and Immunohistochemistry

The liver tissues were harvested from mice and fixed in 4% paraformaldehyde for 24 h. The sections were subsequently embedded in paraffin and stained with H&E. For immunohistochemical analysis, sections were deparaffinized and rehydrated, followed by antigen retrieval. The sections were incubated with peroxidase blocking reagent for 15 min, followed by incubating 3% BSA for 30 min and primary antibody CD11b or CD68 overnight at 4°C. After extensive washing with PBS, the sections were incubated with secondary antibodies for 50 min at room temperature. Sections were washed and stained with substrate-chromogen solution and hematoxylin. All images were acquired by a light microscopy. Corresponding CD11b and CD68 positive cells were calculated with Image J software.

### Alanine Aminotransferase (ALT), Aspartate Transaminase (AST), and Lactate Dehydrogenase (LDH)

Serum ALT, AST, and LDH were determined using the commercially available assay kit (Nanjing Jiancheng Bioengineering Institute, Nanjing, China) according to the manufacturer’s instructions.

### Analysis of Serum Cytokines

IL-1β, IL-18, TNF-α, IL-17, and IL-22 concentrations in serum were assessed using enzyme-linked immune sorbent assay (ELISA) kits (Multi Sciences, Hangzhou, China) according to the manufacturer’s instructions.

### Western Blot

Mice liver tissues were homogenized with RIPA lysis buffer, followed by centrifugation to clear the lysates. The concentrations of protein were then measured by bicinchoninic acid method (BCA) protein assay. Equivalent amounts of total protein (20 µg) were separated on a SDS-PAGE gel and then transferred onto PVDF membranes. Membranes were blocked with 3% BSA for 2 h at room temperature, and then incubated overnight at 4°C with primary antibodies, including NLRP3, Cleaved caspase-1, IL-1β, GAPDH, JAK2, p-JAK2, STAT3, and p-STAT3, followed by secondary antibodies for 2 h at room temperature. Chemiluminescence detection kit (Millipore, Billerica, MA, USA) was applied to visualize the membranes, and densitometric quantification of protein expression was obtained by Image J software.

### Isolation of Primary Hepatocytes and Nonparenchymal Liver Cells

Primary hepatocytes were isolated using a two-step perfusion method. The livers were perfused with 20 ml of D-Hanks buffer and 20 ml of 0.02% collagenase IV solution (Yeasen, Shanghai, China). Liver tissues were minced to dissociate cells followed by filtration through a 200 µm pore mesh. Cell pellet was re-suspended with RPMI-1640 culture medium, and then added to the same volume of Percoll solution (GE Healthcare Life Sciences, Pittsburgh, PA, USA). After centrifuging at 500 *g* for 10 min, cell pellet was re-suspended in culture medium. For the isolation of nonparenchymal cells, the cell pellet was re-suspended with 35% Percoll solution and overlaid on a 70% Percoll solution. After centrifuging at 2,000 rpm for 20 min, the interphase was collected and re-suspended in culture medium.

### Splenocytes Proliferation Assay

Homogeneous spleen cells were isolated by gently mincing the organ in RPMI-1640 culture medium before passing through a 200 µm pore mesh. Splenocytes were seeded in a 96-well culture plate with or without 1 µg/ml ConA, and different concentrations of rhIL-1Ra were pretreated 2 h before ConA incubation. After treatment with rhIL-1Ra for 24 h, 0.5 mg/ml of MTT was added to each well for 4 h followed by 100 µl DMSO dissolving, and the optical density (OD) was measured by Microplate Reader.

### Intracellular ROS, mtROS, and Mitochondrial Membrane Potentials

Reactive oxygen species assay kit (Beyotime Biotechnology, Haimen, China) was used to detect the whole intracellular ROS in cells isolated from ConA-treated mice. Twelve hours after ConA injection, the whole cells were collected and incubated with 10 µM fluorescent probe 20, 70-dichlorofluorescein diacetate (DCFH-DA) in serum-free medium at 37°C for 20 min. Cells were washed three times with serum-free medium and a fluorescence microplate reader was used to determine the OD value (excitation: 488 nm, emission: 525 nm). The level of mtROS and mitochondrial membrane potentials were determined using MitoSox™ Red reagent (Molecular Probes, OR, USA) and JC-1 probe (Beyotime Biotechnology, Haimen, China). Briefly, unfixed frozen liver sections were prepared and incubated with 5 µM MitoSox™ Red or 5 µg/ml JC-1 staining solution at 37°C for 30 min and counterstained with Hoechst 33342 to detect nuclei. All results were visualized by confocal microscopy and relative fluorescence was quantified by Image J software.

### Pyroptosis

Pyroptosis was assessed by double positive staining of FAM-YVAD-FMK and propidium iodide (PI) in frozen liver sections using the FAM-FLICA Caspase-1 Detection Kit (ImmunoChemistry, Bloomington, MN, USA). Twelve hours after ConA injection, unfixed frozen liver sections were stained with green FLICA caspase-1 inhibitor reagent at 37°C for 60 min and counterstained with Hoechst 33342.

### Caspase-1 Activity

Caspase-1 activity was measured by cleavage of chromogenic caspase substrate, Ac-YVAD-*p*NA (Beyotime Biotechnology, Haimen, China). 50 µg of total protein from liver homogenates was added to reaction buffer containing Ac-YVAD-*p*NA, incubated for 2 h at 37°C, and the absorbance of yellow *p*NA was tested using a spectrometer at 405 nm. The caspase-1 activity was normalized for total proteins of cell lysates.

### Immunofluorescence Staining

Twelve hours after ConA injection, the primary hepatocytes were collected and incubated with anti-NLRP3 antibody overnight at 4°C, followed by secondary antibodies and Hoechst 33342 for 1 h at room temperature. The images were taken by confocal microscopy.

### Statistical Analysis

The data were analyzed using GraphPad Prism 5 and presented as mean ± SD. Comparisons of two groups were analyzed using a Student’s *t-*test. * and ** indicated *p* < 0.05 and *p* < 0.01, respectively.

## Results

### NLRP3 Inflammasome Activation and IL-1β Production in ConA-Induced Hepatitis

**Figure 1:**
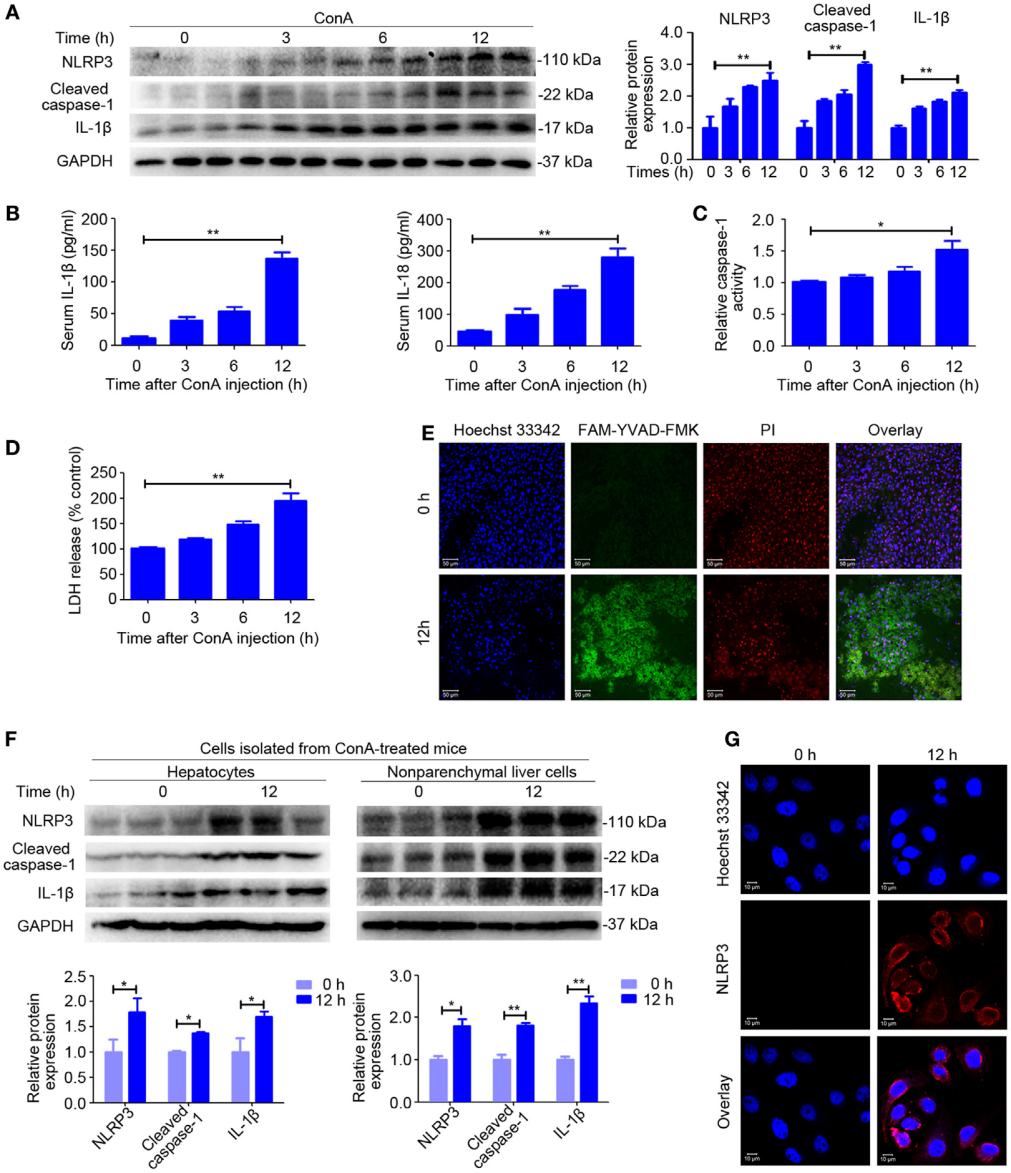
NOD-like receptor protein 3 (NLRP3) inflammasome activation and IL-1β production in ConA-induced hepatitis. **(A)** BALB/c mice (*n* = 6 for each group) were intravenous administrated with ConA (20 mg/kg), and sera and liver tissues were obtained following ConA injection at 0, 3, 6, and 12 h. The expression of NLRP3, Cleaved caspase-1, and IL-1β in the livers were detected by western blot analysis. GAPDH was provided as a loading control. Each lane represented a separate animal. The blots were representative of three experiments. Densitometric values of these proteins were quantified using the Image J software. **(B)** Serum concentrations of IL-1β and IL-18 were analyzed by enzyme-linked immune sorbent assay. **(C)** Caspase-1 enzymatic activity in the liver homogenates was measured. **(D)** Lactate dehydrogenase (LDH) release into serum. **(E)** FAM-YVAD-FMK and propidium iodide (PI) double staining of liver tissues were applied to detect pyroptosis. FAM-YVAD-FMK (green), PI (red), Hoechst 33342 (blue), Scale bars = 50 µm. **(F)** The protein levels of NLRP3 pathways in the primary hepatocytes and nonparenchymal liver cells isolated from ConA-treated mice were detected by western blot analysis. GAPDH was used as a loading control. Each lane represented a separate animal. Results represented three independent experiments. Quantification was presented. **(G)** The expression of NLRP3 in primary hepatocytes was displayed by immunofluorescence. NLRP3 (Red), Hoechst 33342 (blue), scale bars = 10 µm. The data were presented as means ± SD (Student’s *t*-test, *p < 0.05, **p < 0.01).

First, we assessed whether NLRP3 inflammasome and IL-1β were activated in ConA-induced hepatitis, western blot was employed to observe protein expression of NLRP3 inflammasome and IL-1β. A time course data revealed that levels of NLRP3, Cleaved caspase-1, and IL-1β increased in the livers of ConA-treated mice in a time-dependent manner (Figure 1A). Moreover, serum concentrations of IL-1β and IL-18 were also markedly augmented at corresponding time points (Figure 1B). Importantly, ConA-treated mice showed an increase levels of serum LDH in series time and elevated activity of caspase-1 in liver homogenates (Figures 1C,D), which is likely to trigger an inflammatory form regulated cell death known as pyroptosis, characterized by plasma membrane rupture and release of intracellular contents (30). Although pyroptosis was proposed to be responsible for liver defense against bacterial infection, but its role in AIH has never been addressed (31). Therefore, we next took advantage of death cell staining to further investigate whether pyroptosis occurred in this murine model of AIH. As exhibited in Figure 1E, co-staining of liver tissues with PI and FAM-YVAD-FMK, a caspase-1-specific marker, showed that pyroptosis predominantly occurred in livers at 12 h post ConA administration.

In order to identify the cell population responsible for increased production of NLRP3 inflammasome, we isolated the primary hepatocytes and nonparenchymal liver cells from mice exposed to ConA for 12 h and extracted total protein from each cell population. Western blot analysis suggested that both hepatocytes and nonparenchymal liver cells expressed NLRP3, Cleaved caspase-1, and IL-1β, but nonparenchymal liver cells induced a higher level of expression than hepatocytes (Figure 1F). Immunofluorescence of the primary hepatocytes isolated from ConA-treated mice confirmed that NLRP3 protein expression in hepatocytes (Figure 1G).

In summary, our data indicated that NLRP3 inflammasome activation and IL-1β production, as well as pyroptosis strongly increased in ConA-induced hepatitis.

### NLRP3 Deficiency Alleviated ConA-Induced Liver Injury

**Figure 2:**
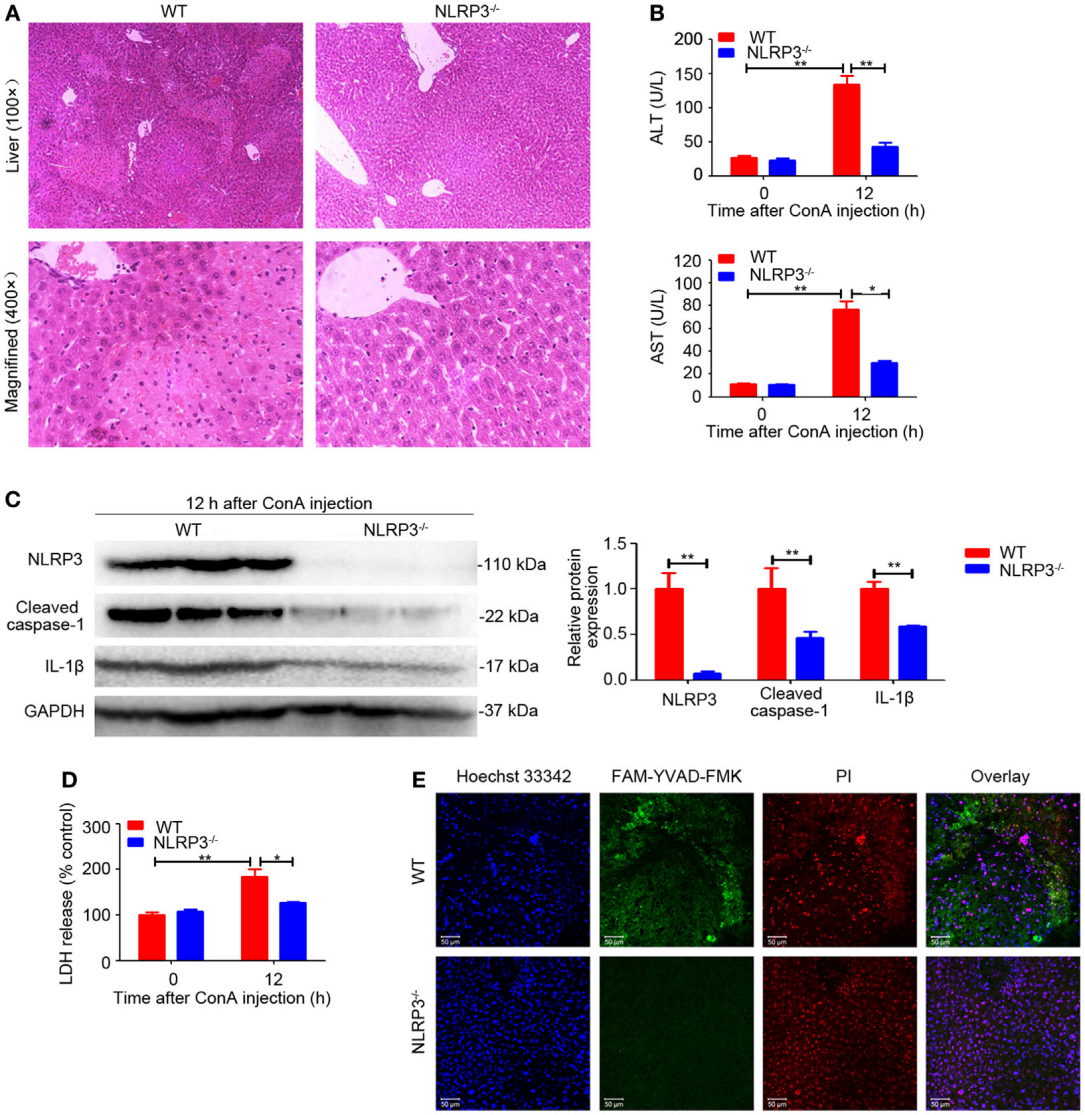
NOD-like receptor protein 3 (NLRP3) deficiency alleviated ConA-induced liver injury. **(A)** *NLRP3^−/−^* mice and WT mice (*n* = 6 for each group) were treated with ConA (20 mg/kg), and corresponding samples were isolated from mice at 0 and 12 h post ConA challenge. Hepatocellular damage was detected by hematoxylin and eosin (H&E) and representative images of liver tissues were shown (100×, magnification: 400×). **(B)** Serum ALT and aspartate transaminase levels in WT and mice were detected. **(C)** The protein levels of NLRP3, cleaved caspase-1 and IL-1β in the livers of mice with autoimmune hepatitis (AIH) were analyzed by western blot. GAPDH was used as a loading control. Each lane represented a separate animal. Results shown were obtained from three experiments. Quantification was presented in bar graphs. **(D)** Pyroptosis was detected by serum lactate dehydrogenase (LDH). **(E)** Representative fluorescence images of liver tissues co-stained with FAM-YVAD-FMK and propidium iodide (PI). FAM-YVAD-FMK (green), PI (red), Hoechst 33342 (blue). The data were presented as means ± SD (Student’s *t*-test, *p < 0.05, **p < 0.01).

To evaluate the functional contribution of NLRP3 in immune-mediated hepatitis, *NLRP3^−/−^* mice and wild-type (WT) mice were exposed to ConA challenge. In sharp contrast to WT mice, *NLRP3^−/−^* mice were almost entirely protected from liver injury as indicated by a significantly reduced hepatocellular damage (Figure 2A) and diminished serum ALT and AST levels after exposure to ConA for 12 h (Figure 2B). In addition, western blot analysis showed that downregulated protein levels of Cleaved caspase-1 and IL-1β were both found in liver homogenates, isolated hepatocytes, and nonparenchymal liver cells of *NLRP3^−/−^* mice (Figure 2C; Figure S4 in Supplementary Material). Notably, NLRP3 deficiency blocked LDH release (Figure 2D) and decreased the number of FAM-YVAD-FMK and PI double-positive cells in the livers (Figure 2E), indicating that pyroptosis was present in mice with ConA-induced hepatitis and could be controlled by knocking out NLRP3. Collectively, these results indicated that NLRP3 played a pathogenic role in the pathogenesis of ConA-induced hepatitis through eliciting IL-1β production and pyroptosis.

### Caspase-1 Deficiency Suppressed ConA-Induced Hepatitis

**Figure 3:**
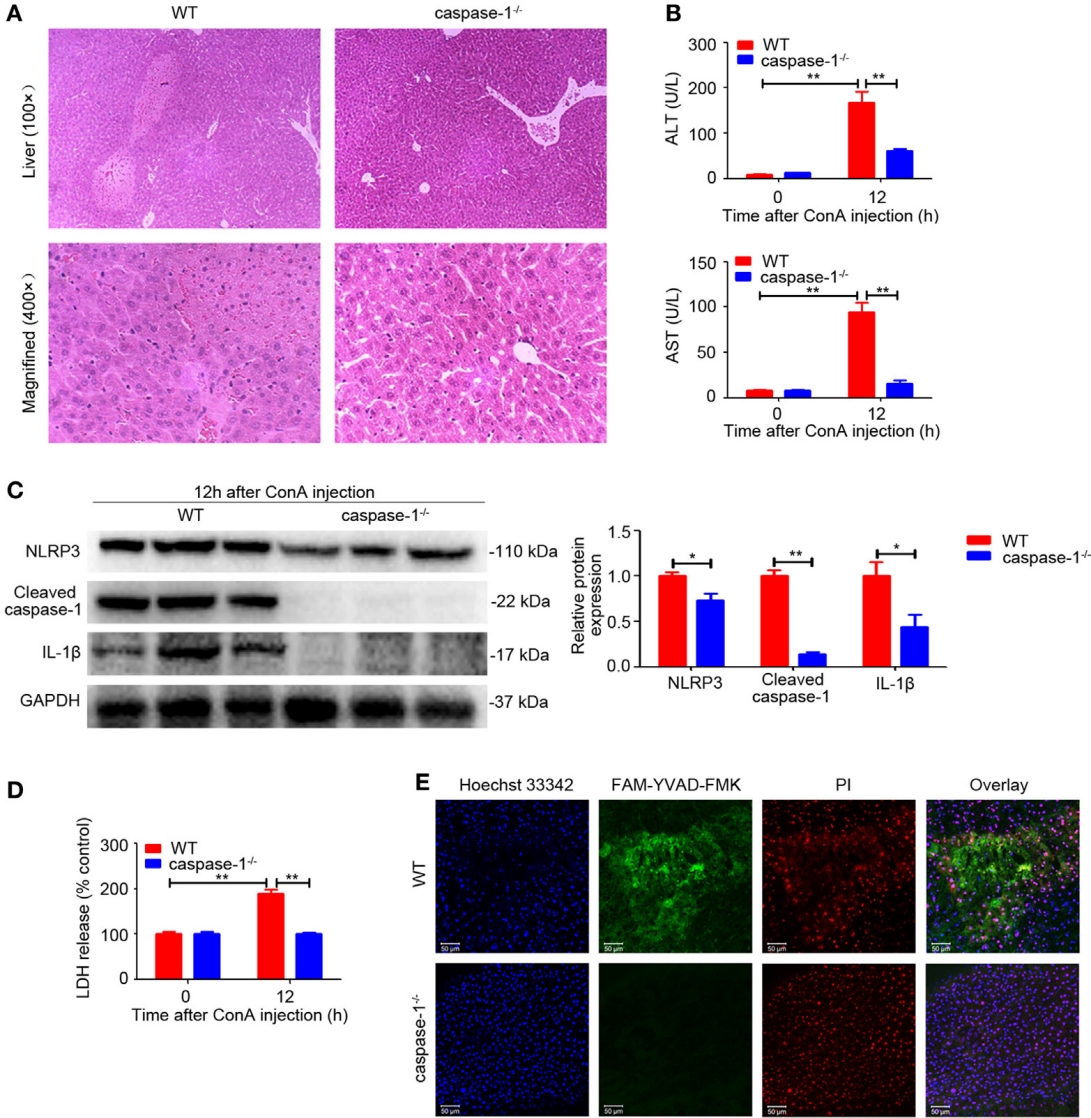
Caspase-1 deficiency suppressed ConA-induced hepatitis. **(A)** *Caspase-1^−/−^* mice and WT mice (*n* = 6 for each group) were subjected to ConA (20 mg/kg) treatment, and samples were collected after exposure to ConA for 12 h. Representative hematoxylin and eosin images of liver tissues were shown (100×, magnification: 400×). **(B)** Serum ALT and aspartate transaminase (AST) levels were analyzed. **(C)** Western blot analysis of NOD-like receptor protein 3, Cleaved caspase-1 and IL-1β in the livers. Densitometric quantification were normalized to GAPDH. Each lane represented a separate animal. Data shown represented three independent experiments. **(D)** Lactate dehydrogenase (LDH) release into serum was measured as signs of pyroptosis. **(E)** Pyroptosis in the livers was observed by confocal fluorescence microscopy. FAM-YVAD-FMK (green), propidium iodide (red), Hoechst 33342 (blue). The data were presented as means ± SD (Student’s *t*-test, *p < 0.05, **p < 0.01).

Given our observation that the expression of Cleaved caspase-1 was also upregulated in the livers of ConA-treated mice (Figure 1A), we next investigated the significance of caspase-1 in regulating IL-1β production. In contrast to WT mice, *caspase-1^−/−^* mice developed ameliorated hepatitis, as exhibited by impaired histological liver injury and decreased serum ALT and AST levels (Figures 3A,B), an outcome similar to that of *NLRP3^−/−^* mice (Figures 2A,B). Furthermore, deficiency of caspase-1 reduced expression of NLRP3, cleaved fragment of caspase-1 and IL-1β in liver tissues (Figure 3C). Meanwhile, LDH release and combined staining of FAM-YVAD-FMK and PI were applied to detect pyroptosis in the livers. As exhibited in Figures 3D,E, both serum LDH and the number of double-positive cells significantly decreased in *caspase-1^−/−^* mice. In brief, these results demonstrated that caspase-1 was required for IL-1β production in ConA-induced hepatitis and caspase-1 deficiency prevented NLRP3 inflammasome activation and pyroptosis, thus ameliorating liver inflammation and injury.

### Blocking IL-1β Protected ConA-Treated Mice From Acute Hepatitis

**Figure 4:**
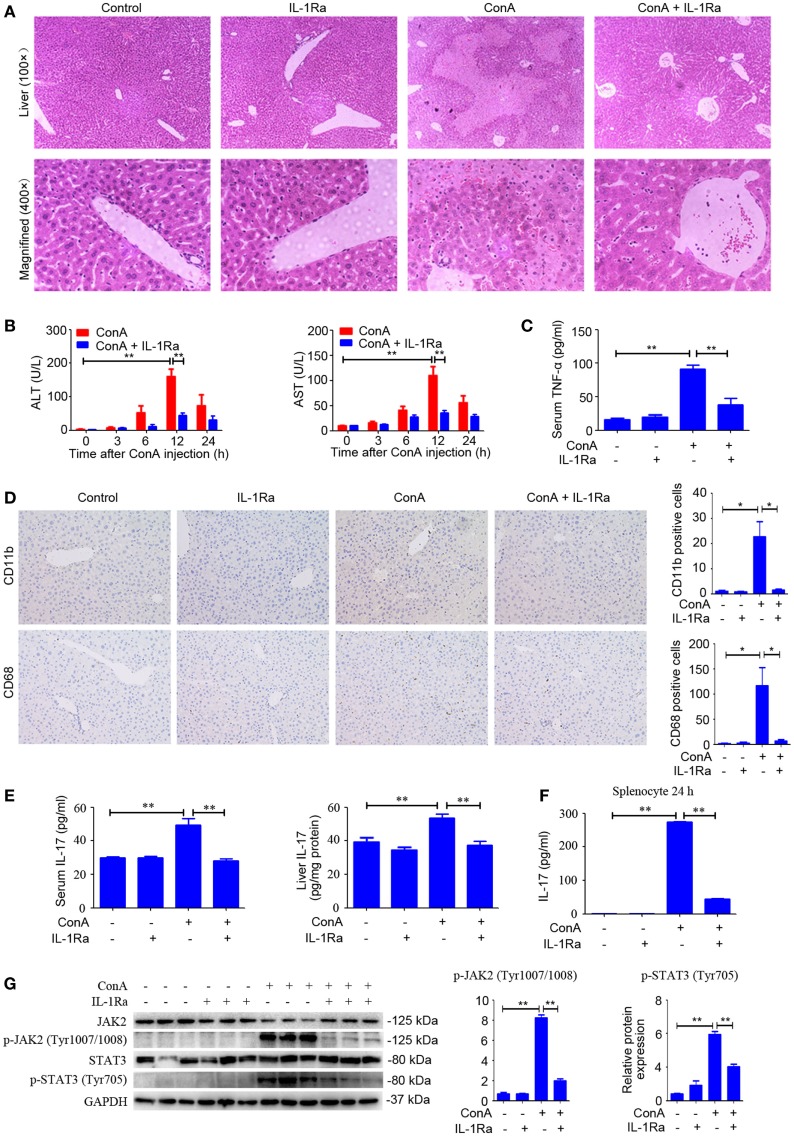
Blocking IL-1β protected ConA-treated mice from acute hepatitis. **(A)** BALB/c mice (*n* = 6 for each group) were pretreated with recombinant human interleukin-1 receptor antagonist (rhIL-1Ra) and subsequently exposed to ConA challenge for 12 h, then sacrificed for collecting samples. Representative H&E images of liver tissues were shown (100×, magnification: 400×). **(B)** Serum ALT and aspartate transaminase (AST) activity were assessed. **(C)** Serum TNF-α was analyzed by enzyme-linked immune sorbent assay (ELISA). **(D)** Representative immunohistochemical images of CD68 or CD11b staining in the liver tissue of mice. Quantification of the number of CD11b-positive cells and CD68-positive cells were obtained in four visual fields (×100) in each group. **(E)** Level of IL-17 in the serum and livers were assessed by ELISA. **(F)** Primary splenocytes were treated with 10 µg/ml rhIL-1Ra in the presence of 1 µg/ml ConA. The concentration of IL-17 in the supernatants was measured by ELISA. **(G)** Western blot was performed to detect JAK2, p-JAK2 (Tyr1007/1008), STAT3, and p-STAT3 (Tyr705) in the livers. Each lane represented a separate animal. The blots were representative of three experiments. Quantification of protein expression with Image J software. The data were presented as means ± SD (Student’s *t*-test, *p < 0.05, **p < 0.01).

NOD-like receptor protein 3 inflammasome activation and caspase-1 cleavage resulted in the maturation and secretion of IL-1β, which was regarded as a critical cytokine in amplifying inflammatory responses (32). RhIL-1Ra, a recombinant human antagonist of interleukin-1 receptor, was employed to block IL-1β in response to ConA. Signs of exaggerated liver injury, including pathological histology, upregulated serum ALT and AST levels, as well as robust TNF-α secretion, were all markedly reduced in rhIL-1Ra-pretreated mice (Figures 4A–C). Moreover, rhIL-1Ra exhibited a suppressive effect on ConA-mediated splenocyte proliferation both *in vitro* and *in vivo* study (Figure S1 in Supplementary Material). In order to detect infiltration of inflammatory cell, CD11b and CD68, were applied to examine inflammatory cells accumulation in the liver. Immunohistochemical analysis showed that the number of CD11b-positive cells and CD68-positive cells significantly increased in the livers, but returned to basal levels by rhIL-1Ra pretreatment (Figure 4D). Additionally, IL-17 levels in serum and liver tissue and JAK2–STAT3 signaling pathway strongly decreased in rhIL-1Ra pretreated group, a finding suggesting that rhIL-1Ra might have a potential suppressive effect on Th17 cells in AIH (Figures 4E,G). To confirm that rhIL-1Ra has a suppressive effect on IL-17, splenocytes were treated with rhIL-1Ra in the presence of ConA for 24 h. ELISA analysis showed that both IL-17 and IL-22 were significantly decreased in rhIL-1Ra-treated group (Figure 4F; Figure S2 in Supplementary Material).

Taken together, these results implied rhIL-1Ra not only reduced inflammatory cell infiltration but also inhibited TNF-α and IL-17 secretion, thus attenuating the pathogenesis of AIH.

### RhIL-1Ra Ameliorated ConA-Induced NLRP3 Inflammasome Activation and Pyroptosis

**Figure 5:**
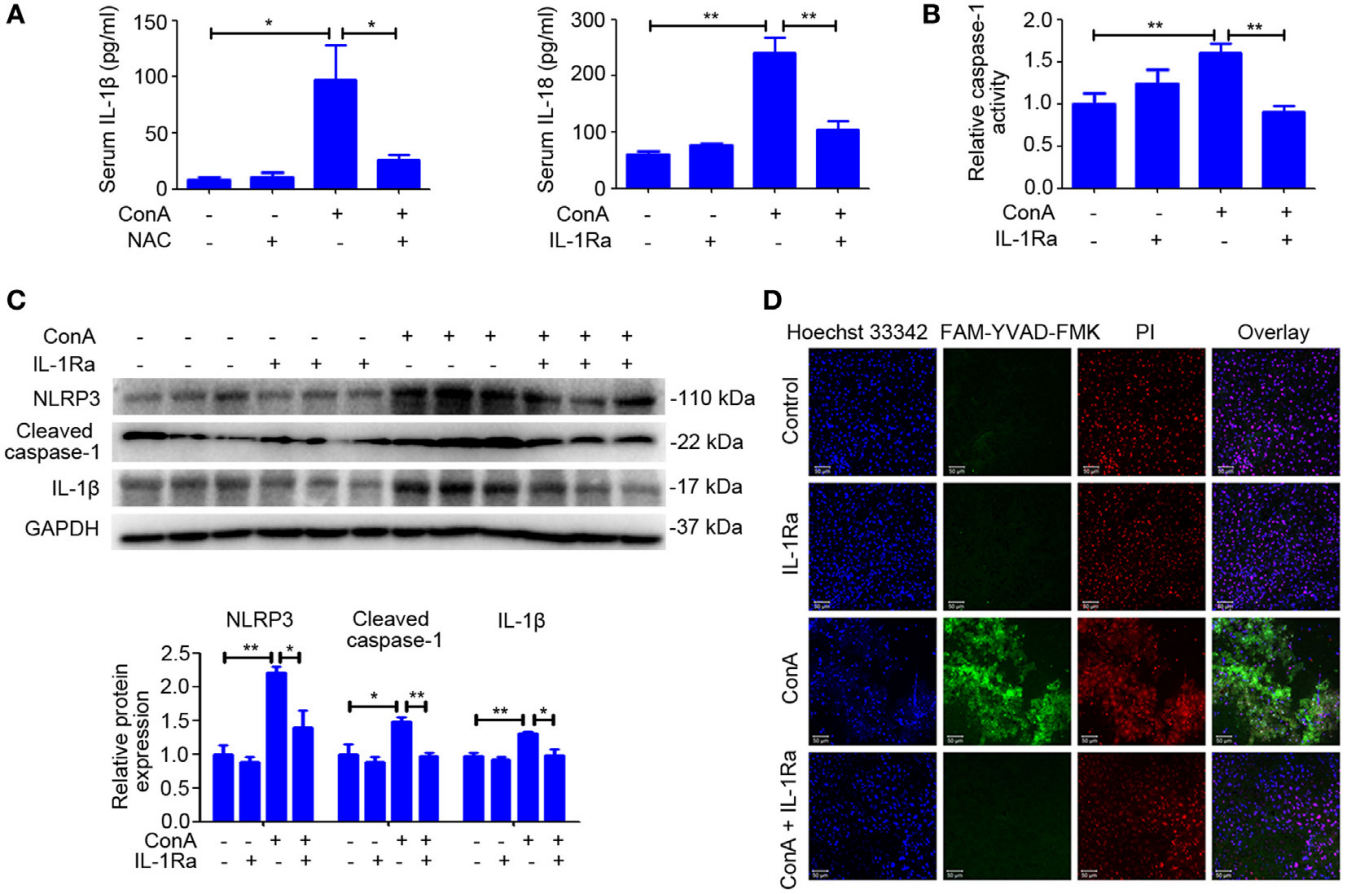
Recombinant human interleukin-1 receptor antagonist (RhIL-1Ra) ameliorated ConA-induced NOD-like receptor protein 3 (NLRP3) inflammasome activation and pyroptosis. **(A)** BALB/c mice (*n* = 6 for each group) were pretreated with rhIL-1Ra before ConA injection, and samples were extracted at 12 h post ConA treatment. Serum IL-1β and IL-18 were detected by ELISA. **(B)** Liver homogenates were subjected to caspase-1 activity assay. **(C)** Western blot of NLRP3, Cleaved caspase-1, and IL-1β in the livers. GAPDH acted as a loading control. Each lane represented a separate animal. Results were obtained from three experiments. Quantification of protein expression were obtained by Image J software. **(D)** Caspase-1 positive death cells staining were observed by confocal microscopy. FAM-YVAD-FMK (green), propidium iodide (red), Hoechst 33342 (blue). The data were presented as means ± SD (Student’s *t*-test, *p < 0.05, **p < 0.01).

Based on the above results, we evaluated whether intervention with rhIL-1Ra could inhibit NLRP3 inflammasome activation. Western blot analysis of NLRP3 inflammasome-related proteins indicated that pretreatment of rhIL-1Ra diminished the expression of NLRP3, Cleaved caspase-1, and IL-1β in liver homogenates, isolated hepatocytes, and nonparenchymal liver cells after ConA challenge for 12 h (Figure 5C; Figure S5 in Supplementary Material). Similar results were observed in serum IL-1β and IL-18 (Figure 5A) as well as the activity of caspase-1 in liver homogenates (Figure 5B). Furthermore, FAM-YVAD-FMK, a green dye that selectively labels caspase-1, was employed to stain liver sections. As revealed by LDH release (Figure S3 in Supplementary Material) and active caspase-1-positive cell staining (Figure 5D), pyroptosis was significantly reduced by rhIL-1Ra pretreatment.

All these results indicated that rhIL-1Ra was beneficial to inhibit NLRP3 inflammasome activation apart from blocking the effects of IL-1β in ConA-induced hepatitis.

### RhIL-1Ra Inhibited NLRP3 Inflammasome Activation *via* Eliminating ROS

**Figure 6:**
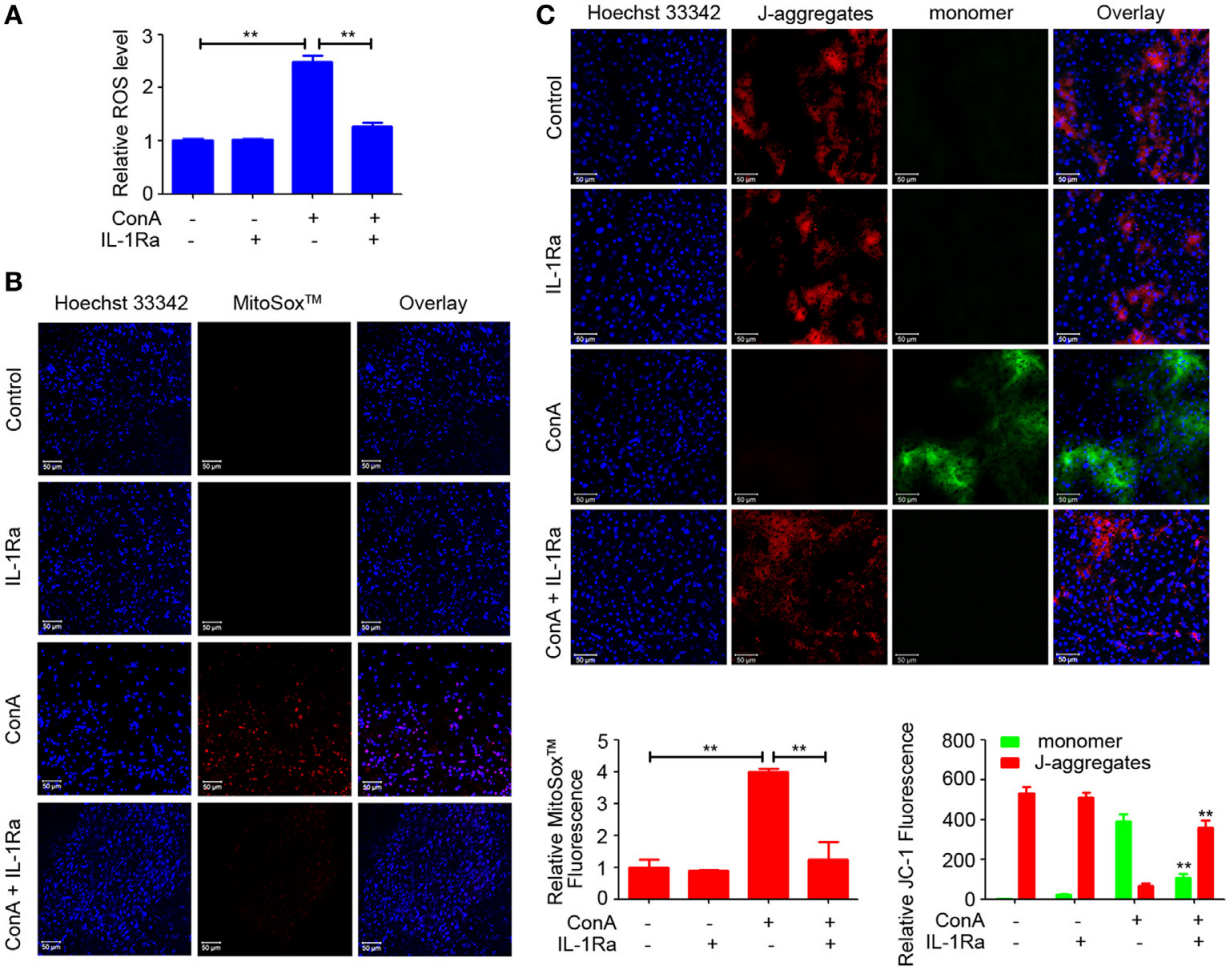
Recombinant human interleukin-1 receptor antagonist (RhIL-1Ra) inhibited NOD-like receptor protein 3 inflammasome activation *via* eliminating reactive oxygen species (ROS). **(A)** BALB/c mice (*n* = 6 for each group) were pretreated with rhIL-1Ra and then intravenously administrated with ConA. 12 h later, samples were collected for following analysis. Intracellular ROS was stained with DCFH-DA (Red) and examined by a fluorescence microplate reader. **(B)** Representative images from liver tissues staining with MitoSox™ were obtained by confocal microscopy. MitoSox™ (Red), Hoechst 33342 (blue), Scale bars = 50 µm. Relative MitoSox™ fluorescence were quantified. **(C)** JC-1 probe was used to detect mitochondrial membrane potentials in the livers. Scale bars = 50 µm. Relative JC-1 Fluorescence was quantified and analyzed by Zeiss LSM710-ZEN 2012 Software. The value of JC-1 monomer in control was set to 1. The data were presented as means ± SD (Student’s *t*-test, *p < 0.05, **p < 0.01).

Recently, ROS generation has been suggested to play a critical role in NLRP3 inflammasome activation (20–22). To explore whether rhIL-1Ra inhibited NLRP3 inflammasome activation was due to eliminating ROS, we detected the release of total ROS with a fluorescent probe DCFH-DA in the cells isolated from ConA-treated mice. As shown in Figure 6A, intracellular ROS induced in mice with AIH was dramatically reduced by rhIL-1Ra. Immunofluorescence analysis further demonstrated that ConA-induced mtROS was also significantly decreased in the livers of rhIL-1Ra-pretreated mice (Figure 6B). To detect whether ROS accumulation was accompanied with mitochondria dysfunction, JC-1 probe was employed to detect mitochondrial membrane potentials. Our results revealed that ConA-challenged mice showed a green fluorescence in the livers, while rhIL-1Ra-pretreated mice showed a red fluorescence, a characteristic indicator of mitochondrial homeostasis (Figure 6C), implying that rhIL-1Ra inhibited ROS generation and mitochondrial dysfunction in the liver tissue.

### NAC-Attenuated ConA-Induced Hepatitis *via* Suppressing NLRP3 Inflammasome Activation

**Figure 7:**
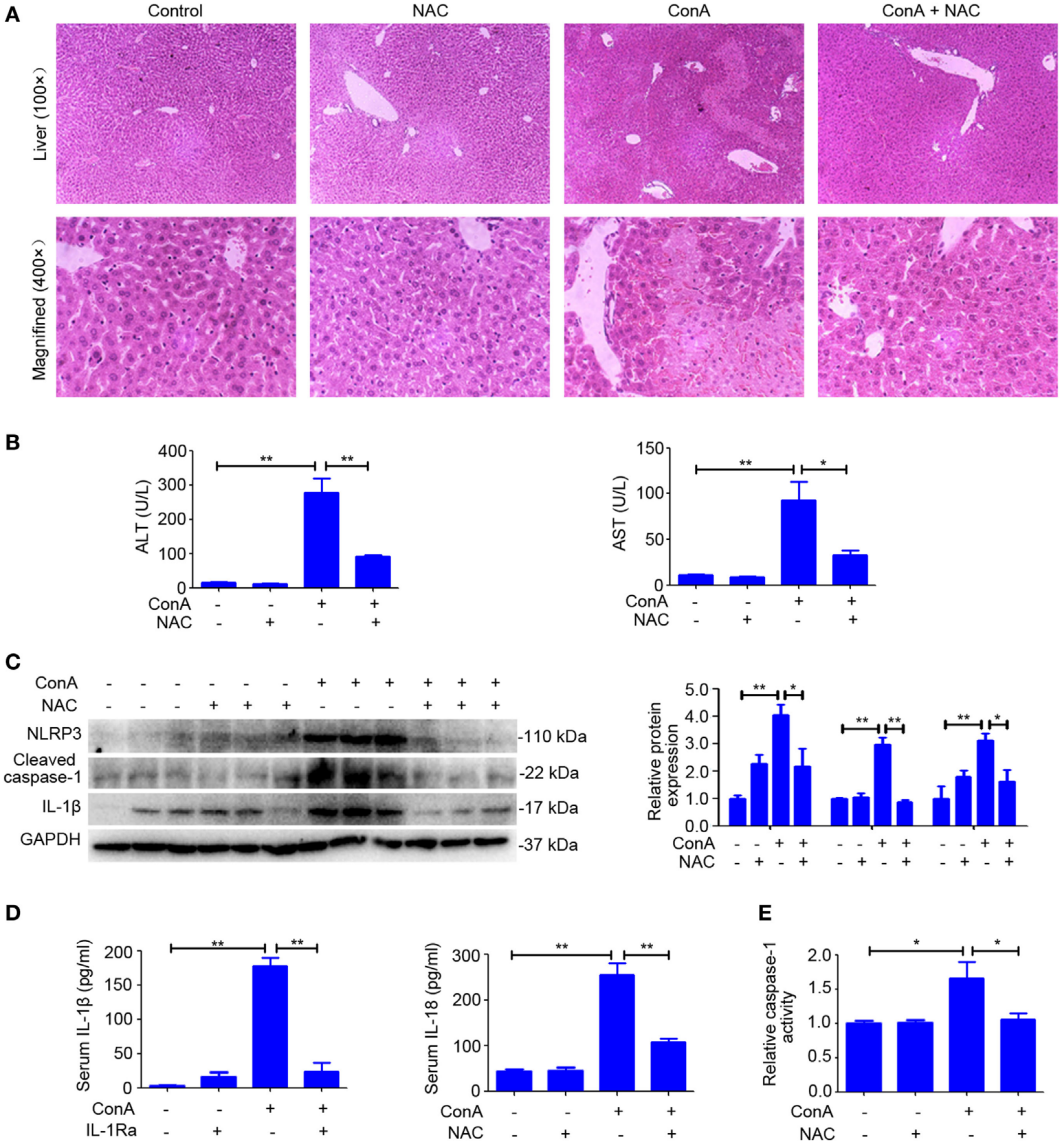
*N*-acetyl-cysteine (NAC) attenuated ConA-induced hepatitis via suppressing NOD-like receptor protein 3 (NLRP3) inflammasome activation. **(A)** BALB/c mice (*n* = 6 for each group) were pretreated with NAC and subsequent exposed to ConA challenge for 12 h, then sacrificed for collecting samples. Representative hematoxylin and eosin images of liver tissues were shown (100×, magnification: 400×). **(B)** Serum ALT and aspartate transaminase (AST) activity were assessed. **(C)** Western blot was performed to detect NLRP3 inflammasome-associated protein NLRP3, Cleaved caspase-1 and IL-1β in the livers. Each lane represented a separate animal. Results shown represented three independent experiments. Quantification of protein expression with Image J software. **(D)** The concentrations of IL-1β and IL-18 in serum were measured by ELISA. **(E)** Caspase-1 enzymatic activity in liver homogenates. The data were presented as means ± SD (Student’s *t*-test, *p < 0.05, **p < 0.01).

Previous publications suggested that ROS contribute to inflammasome activation, which is based on the observation that inhibitors or scavengers that block ROS suppress inflammasome activation (33, 34). To confirm the role of ROS in regulating NLRP3 inflammasome activation in AIH, the scavenger of ROS was pretreated. As anticipated, liver inflammation and injury was significantly reduced by NAC (Figures 7A,B), which also lead to a decrease in the expression of NLRP3, Cleaved caspase-1, and IL-1β in the livers (Figure 7C). In addition, downregulated serum IL-1β and IL-18, as well as dampened activity of caspase-1 in liver homogenates of NAC-pretreated mice indicated that scavenging ROS actually inhibited NLRP3 inflammasome activation (Figures 7D,E). Furthermore, we combined MitoSox™ staining and FAM-YVAD-FMK staining together to detect whether caspase-1 activation occurs in cells where high ROS levels were detected. Immunofluorescence results further demonstrated that ConA-induced ROS accumulation contributed to caspase-1 activation in the liver tissue (Figure S6 in Supplementary Material). Collectively, these results demonstrated that rhIL-1Ra inhibited NLRP3 inflammasome activation in AIH was at least partially dependent on eliminating ROS.

## Discussion

**Figure 8:**
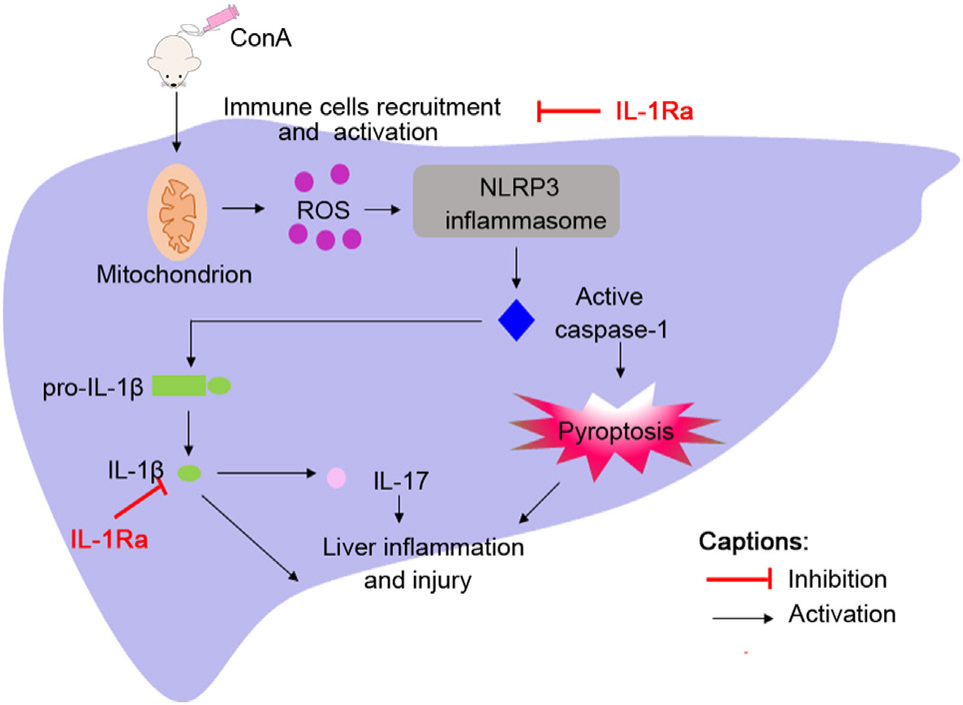
Administration of ConA-induced inflammatory cells infiltration into liver tissues followed by reactive oxygen species (ROS) generation. ROS contributed to NOD-like receptor protein 3 inflammasome activation and caspase-1 cleavage, which elicited IL-1β production and pyroptosis, thus accelerating liver inflammation and injury.

It has been reported that the expression of IL-1β in patients with AIH is significantly increased and correlated with severity of this disease; however, the underlying mechanism of IL-1β activation in the pathogenesis of AIH is still indistinct (7). Here, we reported that NLRP3 and caspase-1 were required for the activation of IL-1β in a murine model of ConA-induced AIH, and mice deficient in NLRP3 or caspase-1 (*NLRP3^−/−^* or *caspase-1^−/−^*) were protected from hepatitis. In addition, we identified the protective role of rhIL-1Ra in ConA-induced hepatitis. Apart from blocking the effects of IL-1β, rhIL-1Ra significantly inhibited NLRP3 inflammasome activation *via* eliminating ROS (Figure 8). Our results provided evidence that NLRP3 inflammasome and IL-1β could be potential targets for the treatment of AIH.

IL-1β is a potent pro-inflammatory cytokine, which stimulates the expression of pro-inflammatory factors and recruits neutrophil to the liver tissue, thus amplifying inflammatory responses (35, 36). Our studies demonstrated that blocking IL-1β by rhIL-1Ra decreased recruitment of CD68-positive cells and CD11b-positive cells into the liver tissue after ConA challenge. Recently, it was reported that IL-1 receptor type 1 (IL-1R1) is critical in the differentiation of Th17 cells, which mediates the progression of autoimmune diseases (37–39). Furthermore, interleukin-23 (IL-23) fails to induce IL-17 in mice deficient in IL-1R and even TNF-α and interleukin-6 (IL-6) enhancement of IL-23-induced IL-17 is likely to be IL-1 dependent. These findings suggest that IL-1β is a central proinflammatory cytokine in inducing IL-23 and IL-6 for Th17 cells differentiation (40–42). Consistent with these findings, we found that decreased secretion of IL-17 in rhIL-1Ra-pretreated splenocytes and decreased production of IL-17 both in serum and livers of rhIL-1Ra-pretreated mice after ConA challenge, which adds new light on the regulation and function of IL-1β on Th17 cells in AIH.

Given that IL-1β functions as a biologically active protein, it remains unclear how IL-1β is processed in experimental AIH. It has proposed that mature IL-1β secretion requires appropriate process by NLRP3 activation and caspase-1 cleavage in various liver diseases, including alcoholic steatohepatitis, viral fulminant hepatitis, non-alcoholic fatty liver disease, and heat stroke-induced liver injury (16–18, 43). Previous studies have indicated the involvement of NLRP3 inflammasome in the development of ConA-induced hepatitis (44–46). Here, we showed that both NLRP3 inflammasome activation and IL-1β secretion were observed in murine model of AIH. And both hepatocytes and nonparenchymal liver cells expressed NLRP3 inflammasome and produce IL-1β. Moreover, *NLRP3^−/−^* and *caspase-1^−/−^* mice displayed downregulated IL-1β expression and diminished hepatitis, indicating that NLRP3 and caspase-1 were essentially required for IL-1β secretion and also contributed to the development of ConA-induced hepatitis in mice. This novel finding opens new perspectives in the pathogenesis and therapy of AIH.

We have determined that NLRP3 inflammasome played an important role in ConA-induced hepatitis, but the mechanism of NLRP3 inflammasome activation has not been demonstrated. Studies have demonstrated that hepatitis virus strain-3, heat stress, and CdSe/ZnS quantum dots triggered NLRP3 inflammasome activation in hepatocytes through inducing ROS generation, which was mainly derived from mitochondria (16, 22, 43). Additionally, a recent study confirmed that ConA increased ROS generation and significantly decreased cell viability (47). Consistent with these studies, our results clearly revealed enhanced mtROS generation and collapse of mitochondrial membrane potential in the liver tissue of mice. While scavenging of ROS by NAC also attenuated liver inflammation, NLRP3 inflammasome activation and IL-1β production, indicating an important role of ROS in mediating NLRP3 inflammasome activation in experimental AIH. An interesting finding in our study was that exogenous IL-1Ra controlled NLRP3 inflammasome activity. RhIL-1Ra, acted as a competitor of IL-1β for IL-1R1 binding, was reported to be beneficial to decrease NLRP3 inflammasome activation apart from blocking the effects of IL-1β (36, 48). Several lines of evidence also confirmed that NLRC4-derived IL-1Ra controlled NLRP3 activation by the ubiquitin/proteasomal degradation pathway in cystic fibrosis (49). We showed here that eliminated ROS and restored mitochondrial membrane function were observed in rhIL-1Ra pretreated mice, which might provide a novel explanation that IL-1Ra inhibited NLRP3 inflammasome was at least partially dependent on eliminating ROS. Collectively, the beneficial effects of rhIL-1Ra on established murine model of AIH were not only due to the blockade of IL-1β, but to the control of NLRP3 inflammasome activity.

Loss of hepatocytes has been regarded as an important mechanism of disease progression in AIH, yet, the precise regulation mechanism of hepatocyte death remains unclear (50). ConA challenge induces an autoinflammatory-mediated liver injury with the infiltration of T lymphocytes to the liver, which was triggered by activation of natural killer T-cells and macrophages, followed by cell death and liver damage (51). Here, in sharp contrast to previous studies suggesting that hepatocyte death was attributed to necrosis, we found that caspase-1-dependent pyroptosis occurred in mice with AIH (52, 53). These findings were further demonstrated in *caspase-1^−/−^* mice, in which liver inflammation was almost prevented, uncovering caspase-1-depedent pyroptosis as a pivotal hepatocyte death in AIH.

In summary, our results identified NLRP3 inflammasome and IL-1β as central mediators in the pathogenesis of ConA-induced hepatitis. Caspase-1-dependent pyroptosis also played a critical role in exacerbating liver injury. Furthermore, the pathogenic NLRP3 inflammasome activity could be ameliorated by rhIL-1Ra. Our findings shed light on the development of promising therapeutic strategies for AIH by blocking NLRP3 inflammasome and IL-1β.

## Ethics Statement

All experimental protocols involving animals were conducted in agreement with the standards approved by Animal Ethical Committee of School of Pharmacy at Fudan University.

## Author Contributions

JL, XZ, and SW designed and performed experiments, analyzed data, and wrote the paper; YL, JF, and WC performed experiments and analyzed data; WZ, SW, and YW analyzed and interpreted data; GM and MC provided intellectual contribution; DJ studied conceptualization, designed experiments, and obtained funding.

## Conflict of Interest Statement

The authors who taken part in this study declared no conflict of interests with respect to this manuscript.
