# Supplementary material for: NOD-Like Receptor Protein 3 Inflammasome-Dependent IL-1β Accelerated ConA-Induced Hepatitis
Source: Front Immunol. 2018 Apr 10;9:758. doi: 10.3389/fimmu.2018.00758 (PMC5902503; doi:10.3389/fimmu.2018.00758)

***Supplementary Material***

**NLRP3 Inflammasome-Dependent IL-1β Accelerated** **ConA-Induced Hepatitis**

Jingyun Luan^1#^, Xuyao Zhang^1#^, Shaofei Wang^1#^, Yubin Li^1^, Jiajun Fan^1^, Wei Chen^1^, Wenjing Zai^1^, Sijia Wang^1^, Yichen Wang^1^, Mingkuan Chen^2^, Guangxun Meng^2^, Dianwen Ju^1*^

^1^Department of Microbiological and Biochemical Pharmacy & The Key Laboratory of Smart Drug Delivery, Ministry of Education, School of Pharmacy, Fudan University, Shanghai, China

^2^Unit of Innate Immunity, Key Laboratory of Molecular Virology & Immunology, Institute Pasteur of Shanghai, Shanghai Institutes for Biological Sciences, Chinese Academy of Sciences, Shanghai, China

^#^These authors contributed equally to this work.

***Corresponding author:** Dianwen Ju, Department of Microbiological and Biochemical Pharmacy & The Key Lab of Smart Drug Delivery, Ministry of Education, School of Pharmacy, Fudan University, Shanghai, 201203, P. R. China; E-mail: dianwenju@fudan.edu.cn; Tel: +86 21 51980037; Fax: +86 21 51980036.

**Supplementary Figure 1.** RhIL-1Ra inhibited ConA-induced splenocyte proliferation. **(A)** BLAB/c mice were pretreated with rhIL-1Ra and followed by ConA challenge for 12 hours, then sacrificed for collecting samples. Spleen index. **(B)** Primary splenocytes were treated with different concentrations of rhIL-1Ra in the presence of 1μg/ml ConA for 24 hours. The data were presented as means ± SD (Student’s *t*-test, * *p* < 0.05, ***p* < 0.01).


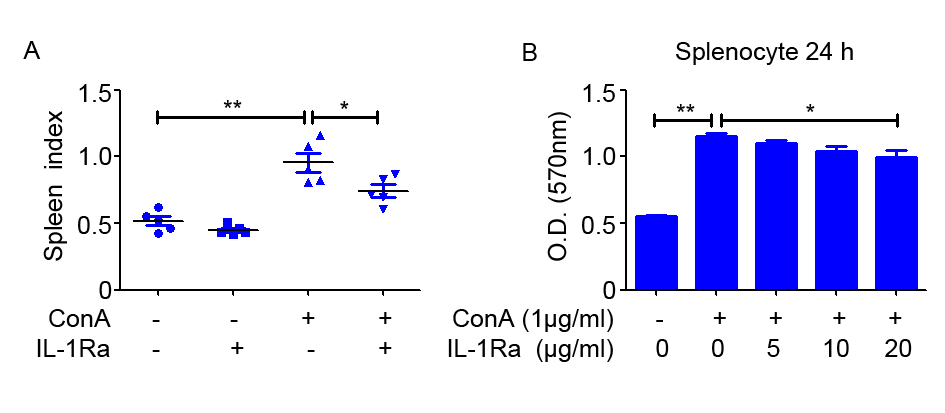


**Supplementary Figure 2.** Treatment with rhIL-1Ra suppressed ConA-induced IL-22 production in splenocytes. Primary splenocytes were treated with 10μg/ml rhIL-1Ra in the presence of 1μg/ml ConA for 24 hours. The concentration of IL-17 in the supernatants was measured by ELISA. The data were presented as means ± SD (Student’s *t*-test, * *p* < 0.05, ***p* < 0.01).


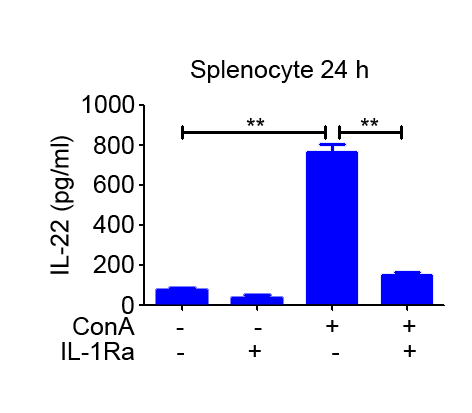


**Supplementary Figure 3.** BALB/c mice were pretreated with rhIL-1Ra before ConA injection, and samples were extracted at 12 h post ConA treatment. Serum LDH was detected. The data were presented as means ± SD (Student’s *t*-test, * *p* < 0.05, ***p* < 0.01).


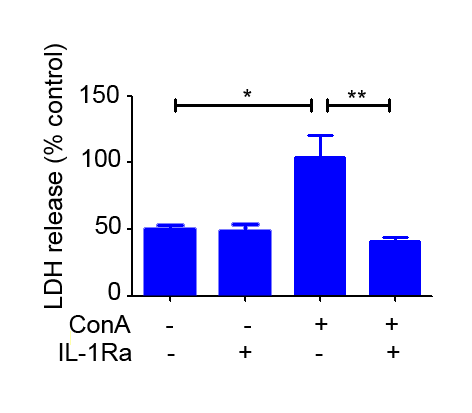


**Supplementary Figure 4**. NLRP3 inflammasome activation was inhibited in hepatocytes and nonparenchymal liver cells in *NLRP3^-/-^* mice. *NLRP3^-/-^* mice and WT mice (n = 6 for all groups) were treated with ConA (20mg/kg), and samples were isolated following ConA injection at 12 h. Protein expression of NLRP3 pathways in the primary hepatocytes and nonparenchymal liver cells were analyzed by western blot. GAPDH acted as a loading control. Each lane represented a separate animal.


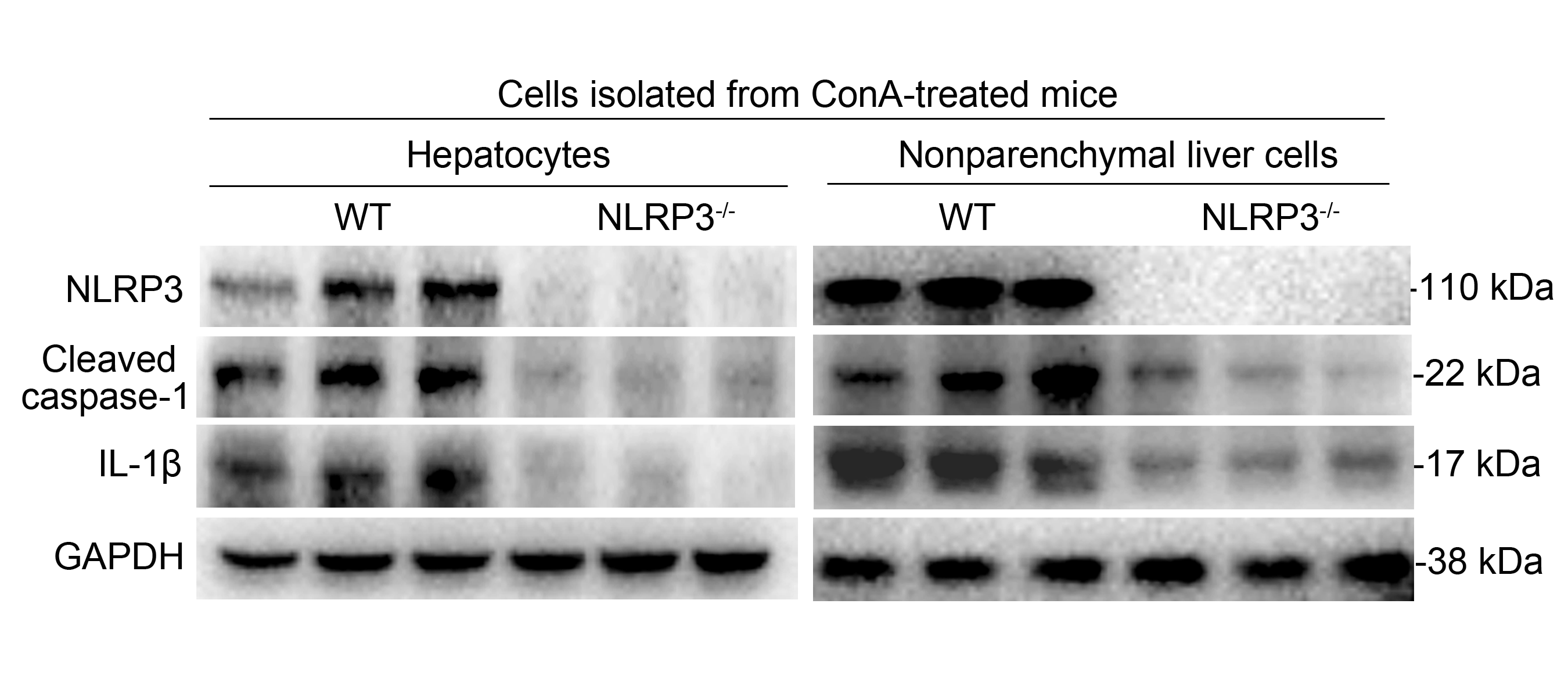


**Supplementary Figure 5**. RhIL-1Ra diminished ConA-induced NLRP3 inflammasome in hepatocytes and nonparenchymal liver cells. BALB/c mice (n = 6 for all groups) were pretreated with rhIL-1Ra and injected with ConA, and samples were isolated and analyzed at 12 h post ConA challenge. The protein levels of NLRP3, Cleaved caspase-1 and IL-1β in the primary hepatocytes and nonparenchymal liver cells were detected by western blot analysis. Each lane represented a separate animal.


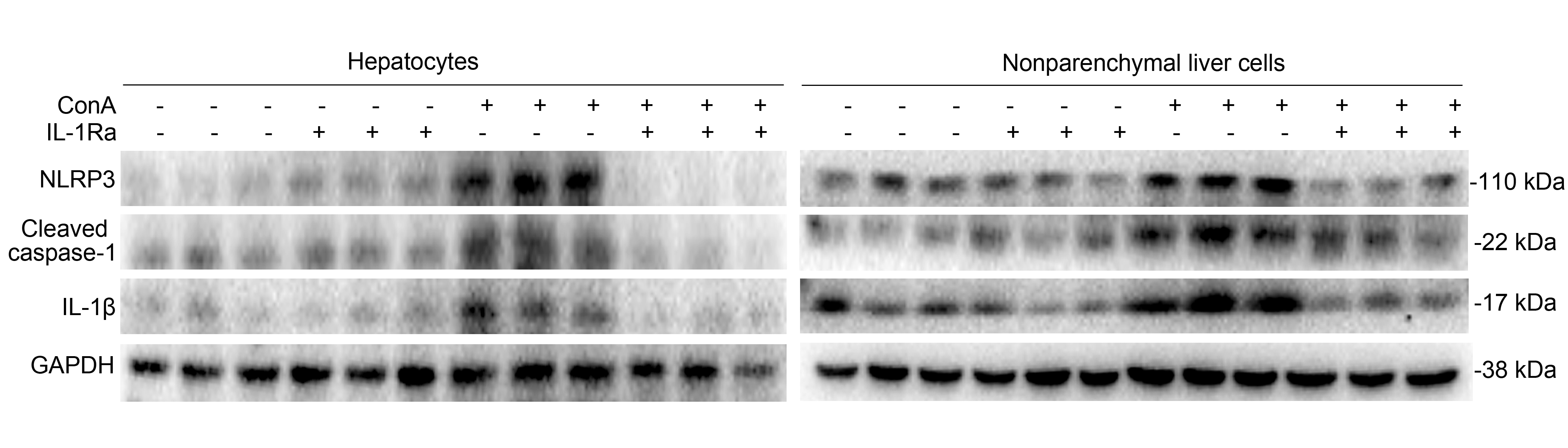


**Supplementary Figure 6.** RhIL-1Ra inhibited ConA-induced ROS and caspase-1. Representative images from liver tissues staining with MitoSox^TM^ and FAM-YVAD-FMK were obtained by confocal microscopy. MitoSox^TM^ (Red), FAM-YVAD-FMK (green), Hoechst 33342 (blue), Scale bars = 10 μm.


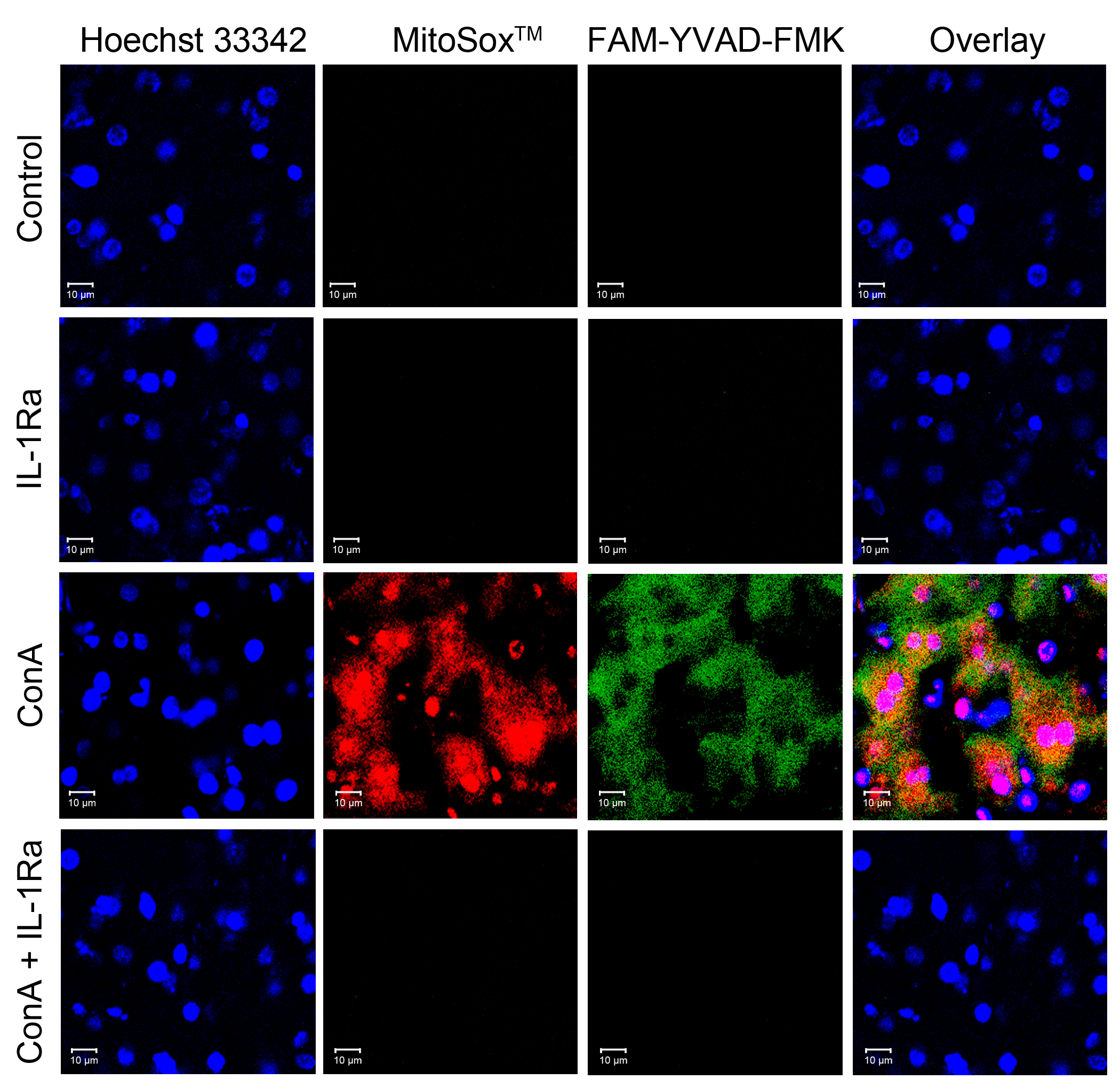

Supplement: Supplementary file 1 [file Data_Sheet_1.DOCX]
